# Mismatch repair deficiency is associated with resistance to DNA minor groove alkylating agents

**DOI:** 10.1038/sj.bjc.6690360

**Published:** 1999-05-01

**Authors:** G Colella, S Marchini, M D'Incalci, R Brown, M Broggini

**Affiliations:** Molecular Pharmacology Unit, Istituto di Ricerche Farmacologiche ‘Mario Negri’, via Eritrea, 62 20157 Milan, Italy; CRC Department of Medical Oncology, Beatson Laboratories, Glasgow G61 1BD, UK

**Keywords:** minor groove binders, mismatch repair, alkylating agents

## Abstract

Mismatch DNA repair deficiency is associated with resistance to certain major groove alkylating agents including methylating agents and cisplatin. We have now studied the relevance of mismatch repair alterations to the cytotoxicity induced by drugs which alkylate N3 adenines in the minor groove of DNA. We have used the mismatch repair defective human colocarcinoma cell line HCT-116 which has a mutation in the hMLH1 gene, and a subline where hMLH1 expression is restored by chromosome 3 transfer (HCT-116+ch3). We have tested three alkylating minor groove binders (tallimustine, carzelesin and CC1065) and one non-covalent minor groove binder (PNU 151807). The HCT-116+ch3 subline was more sensitive than the parental line to the treatment with the three alkylating minor groove binders, while the non-alkylating compound had a similar activity in both cell lines. Further support for mismatch repair being involved in sensitivity of the minor groove alkylators is that two cisplatin-resistant sublines of the human ovarian adenocarcinoma cell line A2780 (A2780/CP70 and A2780/MCP-1) are defective in hMLH1 expression and are more resistant to these agents than the parental mismatch repair proficient cells. Furthermore, the restoration of hMLH1 activity in the A2780/CP70 cell line, by introduction of chromosome 3, was associated with an increased sensitivity to the three alkylating minor groove binders. Again, the non-covalent minor groove binder was equally effective in mismatch repair deficient and proficient clones. The data indicate that mismatch repair deficiency mediated by loss of hMLH1 expression is associated not only with drug-resistance to major groove binders, but also to minor groove binders. However, loss of mismatch repair does not mediate resistance to the non-covalent minor groove binder PNU 151807. © 1999 Cancer Research Campaign


*British Journal of Cancer* (1999) **80**(3/4), 338–343
© 1999 Cancer Research Campaign
Article no. bjoc.1998.0360

### Mismatch repair deficiency is associated with resistance to DNA minor groove alkylating agents

**G Colella[1], S Marchini[1], M D'Incalci[1], R Brown[2] and M Broggini[1]**

[1]Molecular Pharmacology Unit, Istituto di Ricerche Farmacologiche 'Mario Negri', via Eritrea, 62 20157 Milan, Italy; [2]CRC Department of Medical Oncology, Beatson Laboratories, Glasgow G61 1BD, UK

**Summary** Mismatch DNA repair deficiency is associated with resistance to certain major groove alkylating agents including methylating agents and cisplatin. We have now studied the relevance of mismatch repair alterations to the cytotoxicity induced by drugs which alkylate N3 adenines in the minor groove of DNA. We have used the mismatch repair defective human colocarcinoma cell line HCT-116 which has a mutation in the hMLH1 gene, and a subline where hMLH1 expression is restored by chromosome 3 transfer (HCT-116+ch3). We have tested three alkylating minor groove binders (tallimustine, carzelesin and CC1065) and one non-covalent minor groove binder (PNU 151807). The HCT-116+ch3 subline was more sensitive than the parental line to the treatment with the three alkylating minor groove binders, while the non-alkylating compound had a similar activity in both cell lines. Further support for mismatch repair being involved in sensitivity of the minor groove alkylators is that two cisplatin-resistant sublines of the human ovarian adenocarcinoma cell line A2780 (A2780/CP70 and A2780/MCP-1) are defective in hMLH1 expression and are more resistant to these agents than the parental mismatch repair proficient cells. Furthermore, the restoration of hMLH1 activity in the A2780/CP70 cell line, by introduction of chromosome 3, was associated with an increased sensitivity to the three alkylating minor groove binders. Again, the non-covalent minor groove binder was equally effective in mismatch repair deficient and proficient clones. The data indicate that mismatch repair deficiency mediated by loss of hMLH1 expression is associated not only with drug-resistance to major groove binders, but also to minor groove binders. However, loss of mismatch repair does not mediate resistance to the non-covalent minor groove binder PNU 151807.

**Keywords:** minor groove binders; mismatch repair; alkylating agents

Minor groove alkylating agents are a relatively new class of compounds that have shown high anti-tumour activity in experimental systems (Martin et al, 1981; Li et al, 1992; D'Alessio et al, 1994; D'Incalci and Sessa, 1997). The interaction of these compounds with DNA has been studied in detail and it has been found that compounds possessing anti-tumour activity were able to alkylate the N3 of adenines lying in the minor groove with a very high sequence specificity (Hurley et al, 1984; Broggini et al, 1991, 1995). Structure-activity studies on a series of distamycin derivatives have shown that changes in the alkylation potency, without altering the sequence specificity, correlates with the cytotoxicity observed in vitro (Marchini et al, 1997), and that the loss of alkylation ability was associated with loss of in vitro cytotoxicity (Marchini et al, 1997). Recently, PNU 151807, a new derivative of the non-covalent minor groove-interacting compound, distamycin A, has been synthesized. This compound, while able to non-covalently interact with AT-rich regions in DNA, was devoid of any DNA alkylating ability but, interestingly, presented a very high anti-tumour activity both in vitro and in vivo (D'Alessio et al, 1994). The mechanism of action of this compound is not clear, although the non-covalent minor groove interaction seems to be essential at least for the in vivo anti-tumour activity.

*Received 19 March 1998*
*Accepted 27 October 1998*

*Correspondence to:* M Broggini

The promising preclinical activity of this class of compounds has stimulated studies aiming at identifying possible mechanisms of resistance. It has been reported that, in cell lines defective in proteins belonging to the nucleotide excision repair (NER) pathway, tallimustine and CC1065 were only slightly less active than in repair-proficient parental cell lines, while major groove alkylating compounds such as melphalan or *cis*-diammine dichloro platinum (II) (CDDP), were much more active in NER-deficient cell lines (Damia et al, 1996*b*). Moreover, cell lines over-expressing N3-adenine glycosylase had the same susceptibility to tallimustine and CC1065 as parental cell lines (Damia et al, 1996*a*). Proteins belonging to the mismatch repair (MMR) family play an important role in the resistance to alkylating agents and recognition of certain types of major groove DNA damage (Karran and Bignami, 1994; Branch et al, 1993, 1995; Fink et al, 1996; Drummond et al, 1996). Loss of MMR is associated with the development of resistance to monofunctional methylating agents and tolerance to O6-methyl guanine adducts in DNA. Furthermore, cell lines selected for resistance to the bifunctional alkylating agent CDDP were shown to have lost MMR activity (Drummond et al, 1996) and expression of hMLH1 (Brown et al, 1997). Restoration of MMR activity by re-expression of hMLH1 or hMSH2 by chromosome transfer confers increased sensitivity to these agents (Koi et al., 1994; Aebi et al, 1996, 1997). For different major groove alkylating agents, sensitivity of MMR-deficient cell lines was reported to be lower than that observed in mismatch-proficient cell lines (Koi et al, 1994; Anthoney et al, 1996; Aebi et al, 1997; Fink et al, 1997). We report here that minor

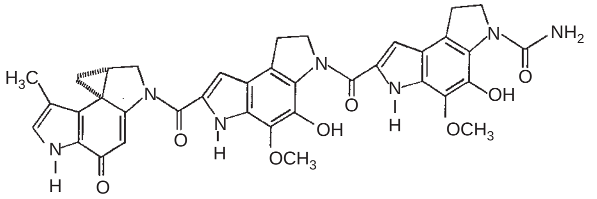

CC-1065

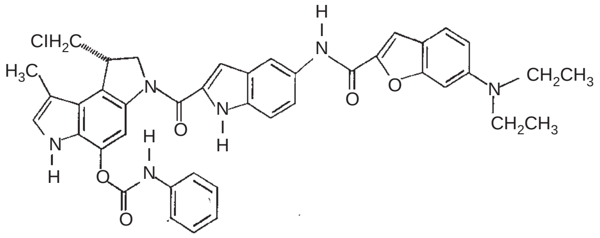

Carzelesin

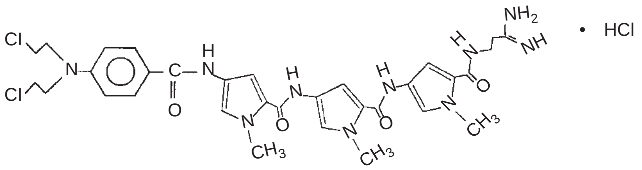

Tallimustine

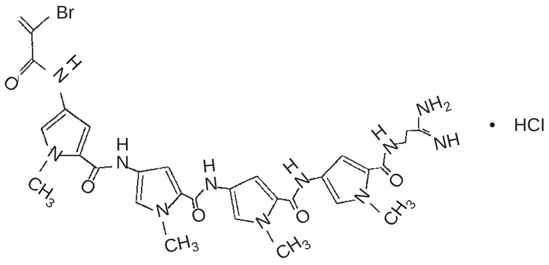

PNU 151807

**Figure 1** Chemical structure of the four minor groove binders groove alkylating agents are also susceptible to the status of MMR and that loss of MMR activity is associated with a decreased activity of all the tested alkylating minor groove binders. The non-alkylating minor groove binder (MGB) PNU 151807 retained its activity irrespective of the MMR status of the cell lines used.

### MATERIALS AND METHODS

### Cell lines

The hMLH1-deficient human colorectal adenocarcinoma cell line HCT-116 and the subline into which a wild-type copy hMLH1 on chromosome 3 has been introduced by microcell fusion (HCT-116+ch3) (Koi et al, 1994) were obtained from Dr G Marra, Zurich. Both cell lines were grown in medium with 400 μg ml$^{-1}$ of G418 (Sigma). The human ovarian cancer cell line A2780 and two CDDP-resistant sublines that have lost hMLH1 expression (Brown et al, 1997) were grown in RPMI-1640 medium (GIBCO) supplemented with 10% fetal calf serum (FCS). A2780/CP70+ch3 clone was obtained from CDDP-resistant, hMLH1-deficient cell line A2780/CP70 by introducing chromosome 3 by microcell-mediated chromosome transfer and was maintained in the same medium as A2780/CP70 cells in the presence of 200 U ml$^{-1}$ of Hygromycin B (Boehringer, Italy).

**Table 1** $IC_{50}$ for the different compounds tested in HCT116 and HCT116+ch3 cell lines

| Compound | HCT116 | HCT116+ch3 | Ratio[a] |
|---|---|---|---|
| CC1065 (pg ml$^{-1}$) | 33.8 ± 1.1 | 10.9 ± 1.0[b] | 3.1 |
| Carzelesin (ng ml$^{-1}$) | 2.6 ± 0.6 | 1.2 ± 0.4[c] | 2.2 |
| Tallimustine (ng ml$^{-1}$) | 59.1 ± 4.7 | 30.1 ± 2.9[b] | 2 |
| PNU 151807 (ng ml$^{-1}$) | 39.6 ± 1.7 | 35.1 ± 1.5 | 1.1 |
| MNNG (μM) | > 25 | 5.0 ± 1.9[b] | > 5 |
| CDDP (μM) | 28.7 ± 3.7 | 12.7 ± 1.8[b] | 2.3 |

The values are the mean ± s.d. of different experiments each consisting of at least three replicates. [a]Ratio between the $IC_{50}$ values of HCT116 and HCT116+ch3. [b]$P \leq 0.01$ and [c]$P \leq 0.05$ vs HCT116.

**Table 2** $IC_{50}$ values for the different compounds in A2780/CP70 and A2780/CP70+ch3 cell lines

| Compound | A2780/CP70 | A2780/CP70+ch3 | Ratio[a] |
|---|---|---|---|
| CC1065 (pg ml$^{-1}$) | 95.5 ± 8.7 | 54.9 ± 8.6[b] | 1.7 |
| Carzelesin (ng ml$^{-1}$) | 5.2 ± 1.3 | 3.8 ± 0.1[c] | 1.4 |
| Tallimustine (ng ml$^{-1}$) | 124.5 ± 16.6 | 47.0 ± 11.0[b] | 2.6 |
| PNU 151807 (ng ml$^{-1}$) | 38.8 ± 2.8 | 40.1 ± 1.9 | 0.9 |
| CDDP | > 100 | 22.1 ± 6.3[b] | > 4 |

The values are the mean ± s.d. of different experiments each consisting of at least three replicates. [a]Ratio between the $IC_{50}$ values of A2780/CP70 and A2780/CP70+ch3. [b]$P \leq 0.01$ and [c]$P \leq 0.05$ vs A2780/CP70.

### Drugs and inhibition of colony formation

For clonogenic assay, cells were plated at 250 cells cm$^{-2}$, treated with different concentrations of compounds for 1 h and incubated for 10–14 days in drug-free medium; the number of colonies formed (roughly 50 cells) were counted after staining with Giemsa.

Student's *t*-test analysis was performed to evaluate differences between parental and chromosome transfected cell lines. $IC_{50}$ values calculated from three independent experiments, each consisting of at least three replicates, were used.

Tallimustine, CC1065, PNU 151807 and carzelesin (see structure in Figure 1) were obtained from Pharmacia & Upjohn (Nerviano, Italy), dissolved as stock solutions in dimethyl sulphoxide (DMSO) and diluted in medium just prior to use. CDDP was obtained from Bristol Myers Squibb and dissolved in medium just prior to use. *N*-methyl-*N*-nitro-*N*-nitrosoguanosine (MNNG) was purchased from Sigma, dissolved in DMSO and subsequently diluted in medium.

*© Cancer Research Campaign 1999*

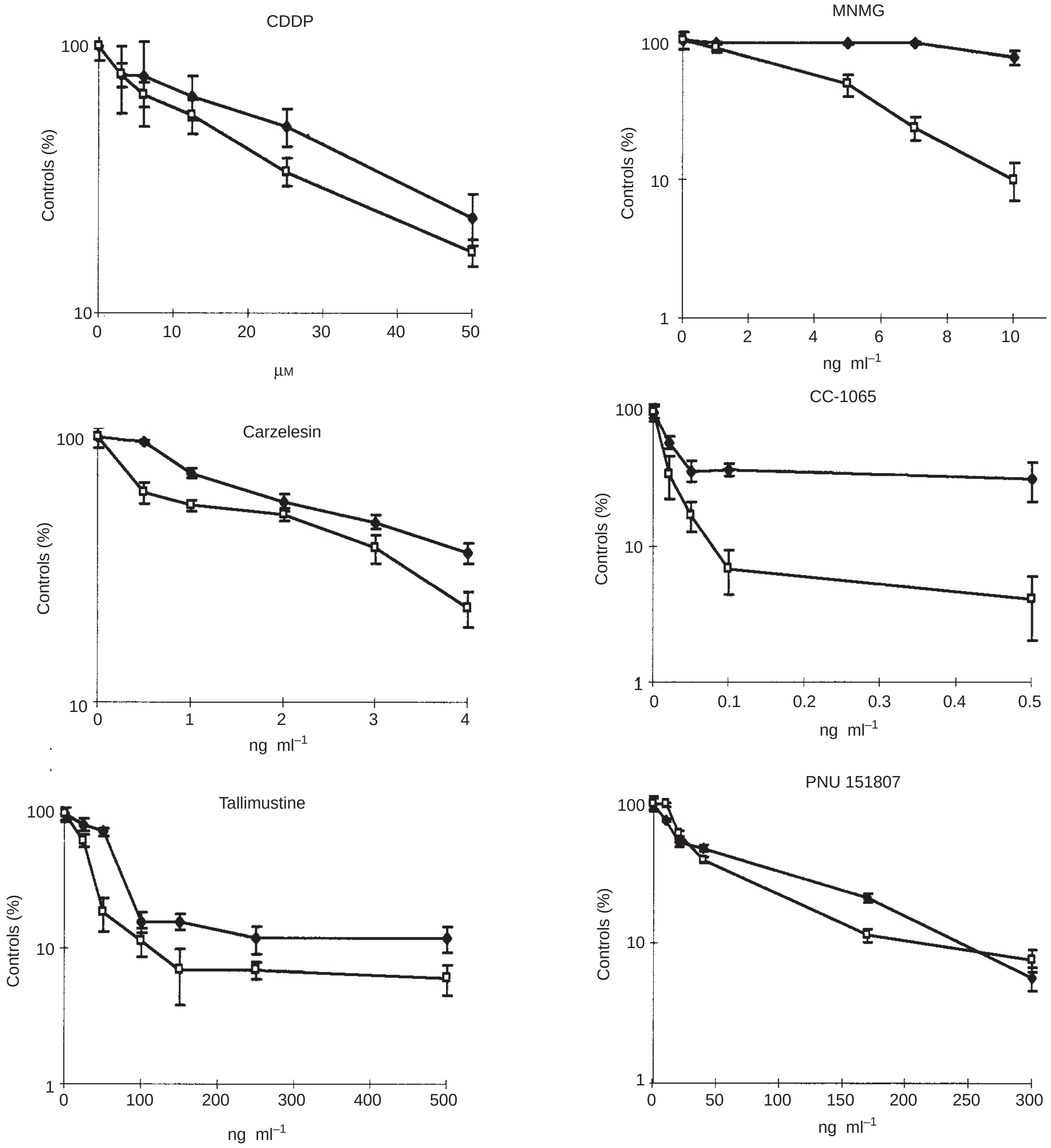

**Figure 2** Effect of DNA mismatch repair deficiency due to loss of hMLH1 function on drug sensitivity determined by clonogenic assay. HCT 116 (◆); HCT 116 + ch3 (□). Each data point represents the mean ± s.d. of three or more experiments performed, each consisting of four replicates

### RESULTS

The cytotoxicity induced by tallimustine, carzelesin, CC1065 and PNU 151807 was evaluated by clonogenic assay in HCT-116 and HCT-116+ch3 cell lines (Figure 2), in comparison with CDDP and MNNG, agents known to exert differential activity in these two cell lines. As shown in Figure 2, the introduction of chromosome 3 in HCT-116 cells results in an increased sensitivity of the cells to the treatment with the three alkylating minor groove binders, but not to the non-covalent MGB PNU 151807, which was equally active in both cell lines. The relative $IC_{50}$ values calculated from the curves shown in Figure 2 are reported in Table 1 where it can be seen that the three alkylating MGB are approximately 2–3 times less active in mismatch-deficient HCT-116 cells than in mismatch-proficient HCT-116+ch3 cells. The difference in activity observed after MNNG treatment was much greater.

The same experiments were performed in another well-characterized system, the human ovarian cancer cell line A2780 (mismatch-proficient) and two sublines, A2780/CP70 and A2780/MCP-1 (obtained as CDDP-resistant clones from A2780 cells), which have been shown to lack expression of hMLH1 protein (Brown et al, 1997). In this system, the three alkylating MGB showed, as in the colocarcinoma cell lines, less activity in the two mismatch-deficient cell lines (Figure 3) and the degree of

 *© Cancer Research Campaign 1999*

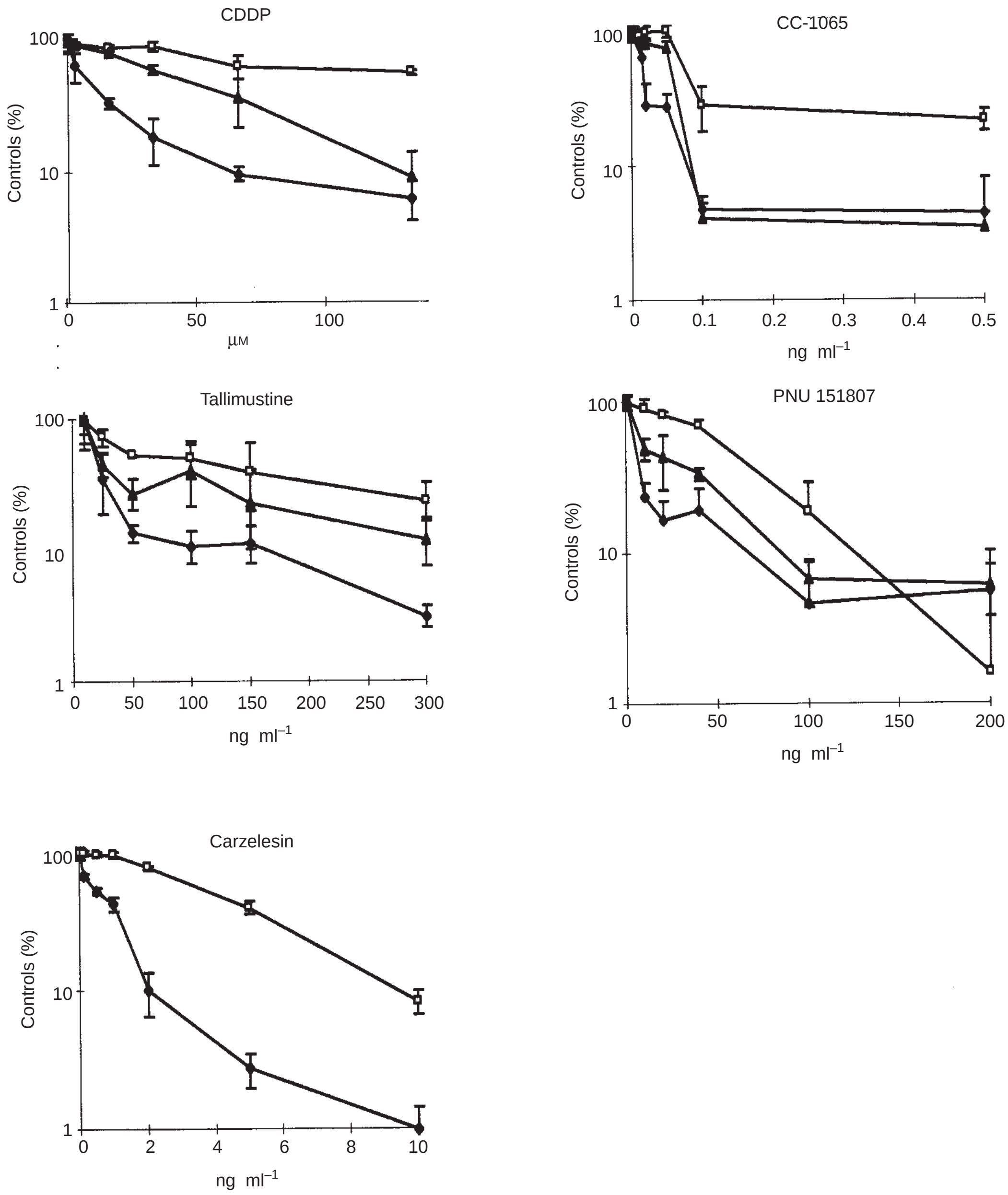

**Figure 3** Clonogenic sensitivities of parental A2780 and mismatch repair-deficient CDDP-resistant subline A2780/CP70 and A2780/MCP1. A2780 (◆); A2780/CP70 (□); A2780/MCP1 (▲). Each data point represents the mean ± s.d. of three or more experiments performed with quadruplicate cultures resistance, calculated from the $IC_{50}$ values, was similar to that found for CDDP (data not shown). The non-alkylating MGB PNU 151807 was also more active in parental A2780 cells than in the two CDDP-resistant clones. As shown in Figure 4 and Table 2, re-expression of hMLH1 in these cells by chromosome transfer partially restores sensitivity to CDDP and the three alkylating MGB, but not to the non-alkylating MGB PNU 151807. A2780/CP70 have previously been shown to have a number of alterations that could affect drug sensitivity. The data presented support a role for MMR in cytotoxicity of the alkylating MGB, but the non-alkylating MGB PNU 151807 retained its activity independently of the mismatch repair status.

### DISCUSSION

The MMR system plays an important role in the control of genomic integrity in cells (Karran and Bignami, 1994; Fishel and Kolodner, 1995; Karran and Hampson, 1996). It has been reported in different experimental systems that defects in MMR proteins confer tolerance to methylating agents and also to some other cytotoxic agents, including CDDP (Anthoney et al, 1996, Fink et al, 1996, 1997). Furthermore, selection of CDDP-resistant cell lines can result in loss of the expression of these proteins (Drummond et al, 1996; Brown et al, 1997). Recently, it has been reported that biopsies of residual disease from human ovarian

© Cancer Research Campaign 1999

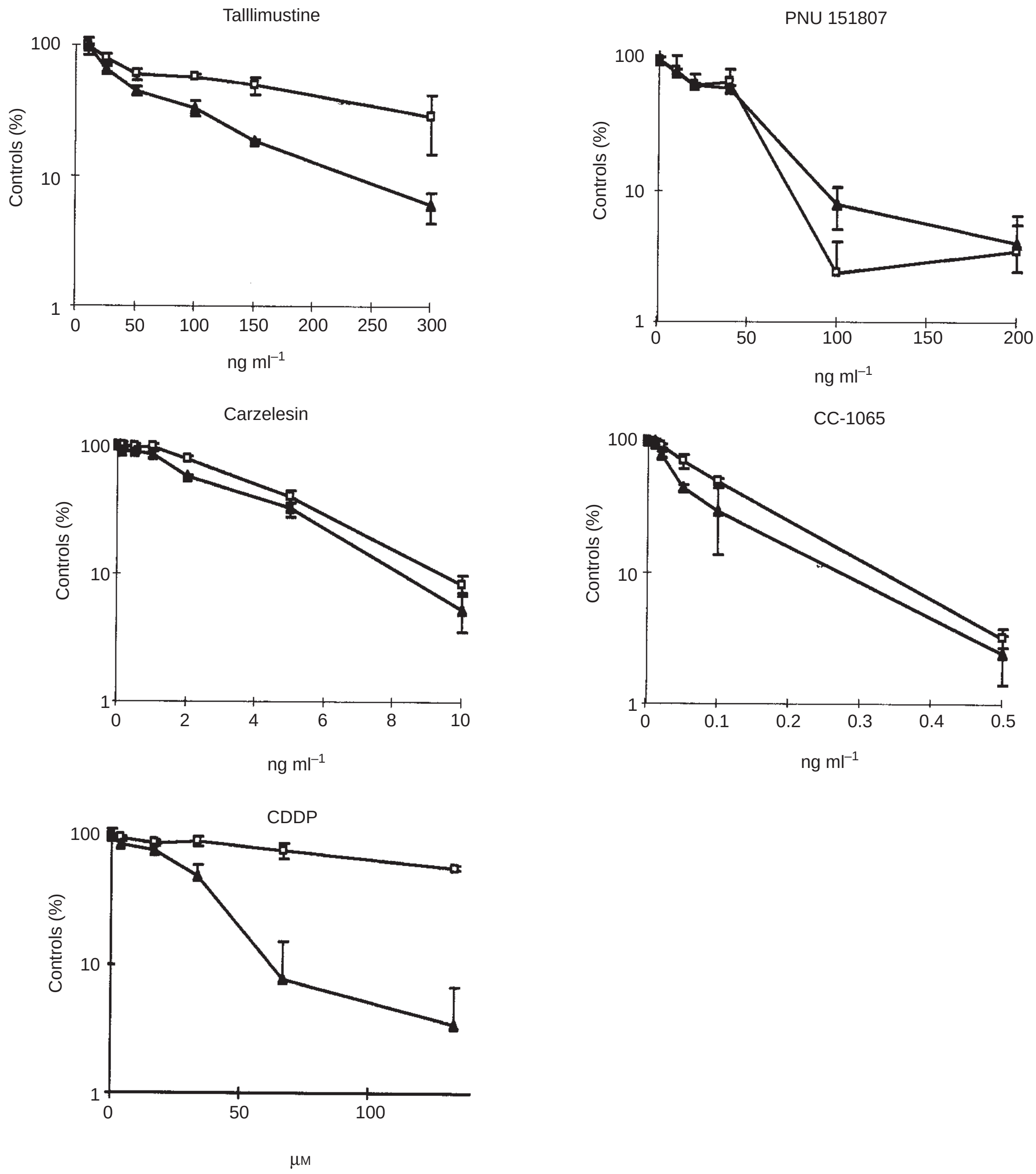

**Figure 4** Clonogenic survival curves in CDDP-resistant ovarian cancer cell lines. A2780/CP70+ch3 (▲); A2780/CP70 (□). Each data point represents the mean ± s.d. of three or more experiments performed with quadruplicate cultures tumours after chemotherapy showed a higher incidence of loss of hMLH1 expression compared to untreated tumours (Brown et al, 1997).

If loss of MMR is indeed relevant for clinical resistance to chemotherapy, this has an important implication for the selection and use of new therapeutic compounds. Thus, compounds not requiring MMR to mediate toxicity will have a different mechanism of action and will have activity in MMR-deficient tumours.

In the present study we show that minor groove alkylating agents, a new emerging class of anticancer agents, also utilize MMR to mediate their tumour cell killing. This is a new finding which increases the number of DNA lesions potentially recognized by the MMR proteins. It should be noted that, in contrast to CDDP which induces a relatively high number of drug adducts in the major groove of DNA, all the three alkylating MGB tested produced only a limited number of DNA lesions (Hurley et al, 1984; Broggini et al, 1991, 1995). This is particularly true for tallimustine which is able to alkylate the N3 of adenine only when present in the hexamer sequence 5′-TTTTGA (Broggini et al, 1995). Despite the different number of DNA lesions induced, the three alkylating MGB showed roughly the same ratio of activity between mismatch-proficient and mismatch-deficient cells observed for CDDP. While it does appear that the MMR-associated resistance does not apply to all the major groove alkylating agents (as reported for example for melphalan, which was able to induce the same cytotoxicity in HCT-116 and

*© Cancer Research Campaign 1999*

HCT-116+ch3 cells) for the minor groove binders, all three compounds tested showed MMR deficiency-associated resistance in both experimental systems used. The non-alkylating MGB PNU 151807 appears to be insensitive to the presence of MMR proteins. This is, in our opinion, an interesting finding, due to the high preclinical anti-tumour activity and the relatively low haematological toxicity shown for this compound (D'Alessio et al, 1994; Ghielmini et al, 1997), and makes it a good future candidate for the treatment of tumours responsive to MGB but presenting alterations in the MMR pathway. As a collateral result we observed that CDDP-resistant cell lines, CP70 and MCP1, are cross-resistant to PNU 151807. However, this resistance is not associated with MMR deficiency, since there is no reversal of resistance in CP70+ch3 cells to the PNU 151807, despite observed reversal resistance for the three alkylating MGB. It must be noted that PNU 151807 is, like tallimustine, a derivative of the non-cytotoxic compound, distamycin A (D'Alessio et al, 1994). Both PNU 151807 and tallimustine showed the same non-covalent DNA interaction, as suggested by footprinting experiments reporting the protection of the same AT-rich region in different DNA fragments (Marchini et al, submitted for publication). The similar activity of PNU 151807 in MMR-proficient and MMR-deficient cells is thus likely to be solely due to the lack of alkylation produced by this compound.

In conclusion, we have shown that some minor groove alkylating agents are processed by the MMR system as has previously been observed for major groove DNA binders. The availability of an active, MMR deficiency-insensitive, non-alkylating anti-tumour agent still binding in the minor groove, could be an important alternative to the clinical use of MGB in MMR-deficient tumours.

### ACKNOWLEDGEMENTS

The generous contribution of the Italian Association for Cancer Research is gratefully acknowledged. RB is supported by the Cancer Research Campaign UK. SM is recipient of a fellowship from the Italian Foundation for Cancer Research (FIRC). We are grateful to Maureen Illand for establishing the A2780/CP70+ chromosome 3 derivatives.

### REFERENCES

Aebi S, Kurdi-Hadar B, Gordon R, Cenni B, Zheng H, Fink D, Christen RD, Boland CR, Koi M, Fishel R and Howell SB (1996) Loss of DNA mismatch repair in acquired resistance to cisplatin. *Cancer Res* **56**: 3087–3090

Aebi S, Fink D, Gordon R, Ki Kim H, Zheng H, Fink JL and Howell SB (1997) Resistance to cytotoxic drugs in DNA mismatch repair-deficient cells. *Clin Cancer Res* **3**: 1763–1767

Anthoney DA, McIlwrath AJ, Gallagher WM, Edlin AR and Brown R (1996) Microsatellite instability, apoptosis, and loss of p53 function in drug-resistant tumor cells. *Cancer Res* **56**: 1374–1381

Branch P, Aquilina A, Bignami M and Karran P (1993) Defective mismatch binding and a mutator phenotype in cells tolerant to DNA damage. *Nature* **362**: 652–654

Branch P, Hampson R and Karran P (1995) DNA mismatch binding defects, DNA damage tolerance, and mutator phenotypes in human colorectal carcinoma cell lines. *Cancer Res* **55**: 2304–2309

Broggini M, Erba E, Ponti M, Ballinari D, Geroni C, Spreafico F and D'Incalci M (1991) Selective DNA interaction of the novel distamycin derivative FCE24517. *Cancer Res* **51**: 199–204

Broggini M, Coley HM, Mongelli N, Pesenti E, Wyatt MD, Hartley JA and D'Incalci M (1995) DNA sequence-specific adenine alkylation by the novel antitumor drug tallumustine (FCE 24517), a benzoyl nitrogen mustard derivative of distamycin. *Nucleic Acids Res* **23**: 81–87

Brown R, Hirst GL, Gallagher WM, McIlwrath AJ, Margison GP, Van Der Zee Age and Anthoney DA (1997) hMLH1 expression and cellular responses of ovarian tumour cells to treatment with cytotoxic anticancer agents. *Oncogene* **15**: 45–52

D'Alessio R, Geroni C, Biasoli G, Pesenti E, Grandi M and Mongelli N (1994) Structure-activity relationship of novel distamycin A derivatives: synthesis and antitumor activity. *Bioorganic Med Chem Lett* **4**: 1467–1472

D'Incalci M and Sessa C (1997) DNA minor groove binding ligands: a new class of anticancer agents. *Exp Opin Invest Drugs* **6**: 875–884

Damia G, Imperatori L, Citti L, Mariani L and D'Incalci M (1996*a*) 3-methyladenine-DNA-glycosylase and O6-alkyl guanine-DNA-alkyltransferase activities and sensitivity to alkylating agents in human cancer cell lines. *Br J Cancer* **73**: 861–865

Damia G, Imperatori L, Stefanini M and D'Incalci M (1996*b*) Sensitivity of CHO mutant cell lines with specific defects in nucleotide excision repair to different anticancer agents. *Int J Cancer* **66**: 779–783

Drummond JT, Anthoney DA, Brown R and Modrich P (1996) Cisplatin and adriamycin resistance are associated with MutLa and mismatch repair deficiency in an ovarian tumor cell line. *J Biol Chem* **271**: 19645–19648

Fink D, Nebel S, Aebi S, Zheng H, Cenni B, Nehmè A, Christen RD and Howell SB (1996) The role of DNA mismatch repair in platinum drug resistance. *Cancer Res* **56**: 4881–4886

Fink D, Zheng H, Nebel S, Norris PS, Aebi S, Lin TP, Nehmè A, Christen RD, Haas M, Macleod CL and Howell SB (1997) In vitro and in vivo resistance to cisplatin in cells that have lost DNA mismatch repair. *Cancer Res* **57**: 1841–1845

Fishel R and Kolodner RD (1995) Identification of mismatch repair genes and their role in the development of cancer. *Curr Opin Genet Dev* **5**: 382–395

Ghielmini M, Bosshard G, Capolongo L, Geroni MC, Pesenti E, Torri V, D'Incalci M, Cavalli F and Sessa C (1997) Estimation of the haematological toxicity of minor groove alkylators using tests on human cord blood cells. *Br J Cancer* **75**: 878–883

Hurley LH, Reynolds VL, Swenson DH, Petzold GL and Scahill TA (1984) Reaction of the antitumor antibiotic CC-1065 with DNA: structure of a DNA adduct with DNA sequence specificity. *Science* **226**: 843–844

Karran P and Bignami M (1994) DNA damage tolerance, mismatch repair and genome instability. *Bioessays* **16**: 833–839

Karran P and Hampson R (1996) Genomie instability and tolerance to alkylating agents. *Cancer Surv* **28**: 69–85

Koi M, Umar A, Chauhan DP, Cherian SP, Carethers JM, Kunkel TA and Boland CR (1994) Human chromosome 3 corrects mismatch repair deficiency and microsatellite instability and reduces *N*-methyl-*N*′-nitro-*N*-nitrosoguanidine tolerance in colon tumor cells with homozygous hMLH1 mutation. *Cancer Res* **54**: 4308–4312

Li LH, Dekoning TF, Kelly RC, Krueger WC, McGovren JP, Padbury GE, Petzold GL, Wallace TL, Ouding RJ, Prairie MD and Gebhard I (1992) Cytotoxicity and antitumor activity of carzelesin, a prodrug cyclopropylpyrroloindole analogue. *Cancer Res* **52**: 4904–4913

Marchini S, Cozzi P, Beria I, Geroni C, Capolongo L, D'Incalci M and Broggini M (1998) Sequence specific alkylation of novel tallimustine derivatives. *Anti-Cancer Drug Design* **13**: 193–205

Martin DG, Biles C, Gerpheide SA, Hanka LJ, Krueger WC, McGovren JP, Mizsak SA, Neil GL, Stewart JC and Visser J (1981) CC-1065 (NSC 298223), a potent new antitumor agent improved production and isolation, characterization and antitumor activity. *J Antibiot Tokyo* **34**: 1119–1125

© Cancer Research Campaign 1999 
